# Methylation of the genes ROD1, NLRC5, and HKR1 is associated with aging in Hainan centenarians

**DOI:** 10.1186/s12920-018-0334-1

**Published:** 2018-02-02

**Authors:** Qian Zeng, Xiaoping Chen, Chaoxue Ning, Qiao Zhu, Yao Yao, Yali Zhao, Fuxin Luan

**Affiliations:** Hainan branch of PLA General Hospital, Sanya, 572000 China

**Keywords:** DNA methylation, Aging, ROD1, NLRC5, HKR1

## Abstract

**Background:**

Human aging is a hot topic in biology, and it has been associated with DNA methylation changes at specific genomic sites. We aimed to study the changes of DNA methylation at a single-CpG-site resolution using peripheral blood samples from centenarians.

**Methods:**

Using Illumina 450 K Methylation BeadChip microarray assays, we carried out a pool-based, epigenome-wide investigation of DNA methylation of blood samples from 12 centenarians and 12 healthy controls. Differentially methylated cytosine-phosphate-guanosine (CpG) sites were selected for further pyrosequencing analysis of blood samples from 30 centenarians and 30 healthy controls.

**Result:**

We identified a total of 31 high-confidence CpG sites with differential methylation profiles between the groups: 9 (29%) were hypermethylated and 22 (71%) were hypomethylated in centenarians. It was also found that hypermethylation of HKR1 and hypomethylation of ROD1 and NLRC5 genes strongly correlated with age in centenarians.

**Conclusion:**

Our results indicate that the methylation profile combination of HKR1, ROD1, and NLRC5 could be a promising biomarker for aging in Hainan centenarians.

**Electronic supplementary material:**

The online version of this article (10.1186/s12920-018-0334-1) contains supplementary material, which is available to authorized users.

## Background

Improvements in living conditions have meant that the average life expectancy of human beings is rising. Indeed, it has increased by approximately 10 years over the last century [1]. On the other hand, aging has attracted more and more attention. Aging is a very complex trait, involving environmental, genetic, and stochastic factors [2], and, in recent decades, has been a focus for scientists worldwide. Centenarian cohort is one of the most valuable models to study the mechanisms involved in human aging [3]. Centenarians are considered to have reached the extreme limits of the human life span, but still remain in relatively good health maintaining physiological functions and avoid succumbing to common fatal diseases [4, 5]. Hainan, with a centenarian density of 21.46 per 100,000 people, is one of the most longevous regions in China. We managed to recruit 42 centenarians and 42 healthy adult controls from Hainan Province, and performed complete physical examinations on all subjects.

Human aging has largely been driven by extrinsic improvements to living conditions, including improved diet and reduced disease prevalence, as well as by both genetic and epigenetic factors [6–8]. Here, we evaluated the crucial role of epigenetic modification in gene regulation, with a particular focus on DNA methylation [9–11]. As early as 1975, it was proposed that DNA methylation could regulate gene activity. Methylation occurs primarily at CpG dinucleotides, which can form CpG islands containing an above average CpG density [12, 13]. DNA methylation has been shown to regulate gene expression and plays a role in alternative silencing [14, 15]. Furthermore, CpG islands overlap the transcription start sites (TSSs) of a majority of human genes, and TSS methylation is known to prevent transcriptional initiation [16, 17]. Yet, the role of methylation in the gene body is less clear. Recent studies have demonstrated the presence of aging-associated CpG sites, which were either hypermethylated or hypomethylated [18–21]. Human genomic methylation has recently been advanced by utilization of the Illumina Human Methylation 450 K BeadChip Array (450 K array), which includes probes spanning 99% of human genes, evenly distributed across the genome. This system can detect the shores and shelves of CpG islands and non-CpG islands, as well as methylation sites in promoter regions, gene bodies, and untranslated regions (UTRs) [22–24].

In the present study, we aimed to identify DNA methylation sites by exploring differences in genomic methylation between centenarians and healthy controls. Using microarray technology and pyrosequencing and a pool-based strategy, we performed an epigenome-wide investigation of DNA methylation in a total of 42 centenarians from Hainan Province. We found that ROD1, NLRC5, and HKR1 genes were differentially methylated between centenarians and healthy controls, suggesting they might be associated with aging in Hainan centenarians.

## Methods

### Subjects

This study formed part of the “Health Investigation and Longevous Mechanism Study on Centenarians in Hainan Province” project, conducted between 2014 and 2016. The subject consisted of 42 centenarians (male: 15, female: 27; Han nationality) and 42 healthy adult controls (male: 18, female: 24; Han nationality). Characteristic information on centenarians and healthy controls is described in Table 1. Significant differences in characteristic information between centenarians and healthy controls are age-related. Healthy controls were excluded if they were suffering from known infectious diseases, or if they had a history of cancer, cardiovascular disease, or autoimmune disease. Neither the centenarians nor the healthy controls included in this study had any infections, and neither group had received any vaccinations in the 15 days prior to the collection of blood samples. Peripheral blood samples were collected by morning fasting. Additional informed consent was obtained from all individual participants for whom identifying information is included in this article.

**Table 1:** Physical characteristics measures of the centenarians (*n* = 42) and healthy controls (n = 42)

Characteristics	Centenarians(n = 42)	Healthy controls(n = 42)
Age (yrs) *	102.2 ± 0.3	49.1 ± 0.8
Height (cm) *	148.3 ± 1.4	166.2 ± 1.2
Mass (kg) *	41.7 ± 1.3	62.0 ± 1.1
BMI (kg/m^−2^) *	18.9 ± 0.5	22.4 ± 0.2
waist circumference (cm)	76.8 ± 1.7	79.8 ± 0.9
Hip circumference (cm) *	86.0 ± 1.2	91.9 ± 0.5

Significant difference from centenarians indicated by * (*p* < 0.05). Data are mean ± SEM

### Genomic DNA extraction

Peripheral blood samples were collected in EDTA-treated collection tubes by experienced nurses during a home visit, and then transferred to the Hainan branch of PLA General Hospital laboratory for processing within 4 h. Genomic DNA was extracted using a QIAamp DNA Mini kit (Qiagen, Germany), according to the manufacturer’s instructions for the Spin Procedure. DNA was eluted in 60 μL of AE elution buffer, and then stored at − 20 °C until use. The concentration and purity of DNA samples were assessed using a Qubit dsDNA HS Assay kit (Invitrogen, Eugene, OR, USA) according to the manufacturer’s instructions, and 1% agarose gel electrophoresis.

### Microarray analysis

Infinium Human Methylation 450BeadChips (Illumina) were used to assay genome-wide DNA methylation. The microarray chip contained 485,577 locus-specific CpG sites. Bisulfite conversion of DNA samples was done using the EZ DNA Methylation Kit (Zymo Research). Microarrays were normalized by methylumi Bioconductor [25–27]. Differentially methylated CpGs were selected using an algorithm in IMA Bioconductor. Probes were used in the analysis, after excluding those with a *P* value higher than 0.01 in more than 25% of the samples, those in chromosome Y and SNP sites. In this study, we assessed the mean-difference *β*-value (Δ*β*) between the two sample groups for each CpG site. The Illumina platform shows a significant dye bias (probe-type design differences) in the two channels which will lead to bias in the estimates of Beta on the GoldenGate platform. Therefore, some normalization was required. Basically, it looks at the median intensities in the methylated and unmethylated channels (each measured in one color on the GoldenGate platform) at very low and very high beta values and sets these medians equal. And then a quantile approach was used to eliminate the batch effects among every microarray. In microarray data analysis, false positives are loci that are found to be statistically different between conditions. However, clinical samples may have relatively high individual heterogeneity, and the amount of samples is not enough. If we use *p*-values for multiple test corrections, it eliminated the false positive, but may have a certain false negative at the same time. Therefore, we considered a probe as differentially methylated if the absolute Δ*β* was higher than 0.2 and the statistical test was significant (*p* value < 0.01) with wilcox [28–30].

### Bioinformatic analysis

As described above, genome-wide DNA methylation was assessed using a pool-based approach, in which the *β*-value for each probe represents the average methylation level of all samples in the pool. To identify differentially methylated probes (DMPs), it was assumed that the genome-wide methylation conformed to a Gaussian model [31], and then differences in methylation between the two data sets were calculated. Gene ontology (GO) and Kyoto Encyclopedia of Genes and Genomes (KEGG) pathway enrichment analyses were conducted using the DAVID database. GO terms and KEGG information were downloaded from the Bioconductor site.

### Bisulfite pyrosequencing analysis

Based on the results of the methylation microarrays, three CpG sites were selected for further pyrosequencing analysis, using blood samples from 30 centenarians and 30 healthy controls. Bisulfite modification of 1-2 μg DNA was performed using an EpiTect Bisulfite kit (Qiagen) according to the manufacturer’s instructions. Next, bisulfite pyrosequencing primers were designed using PyroMark Assay Design software version 2.0 (Qiagen). Sequencing reactions and methylation level quantification were then performed using the PyroMark Q96 ID system and software (Qiagen), respectively. Pyrosequencing primer sequences are presented in additional file 1.

### Statistical analyses

SPSS software version 17.0 (SPSS Inc., Chicago, IL, USA) was only used for the pyrosequencing analyses. Differences between two independent groups were compared using Student’s *t*-tests and *P* < 0.01 was used to define statistical significance.

## Results

### Differential analysis of the DNA methylomes of Hainan centenarians and healthy controls

**Fig. 1:**
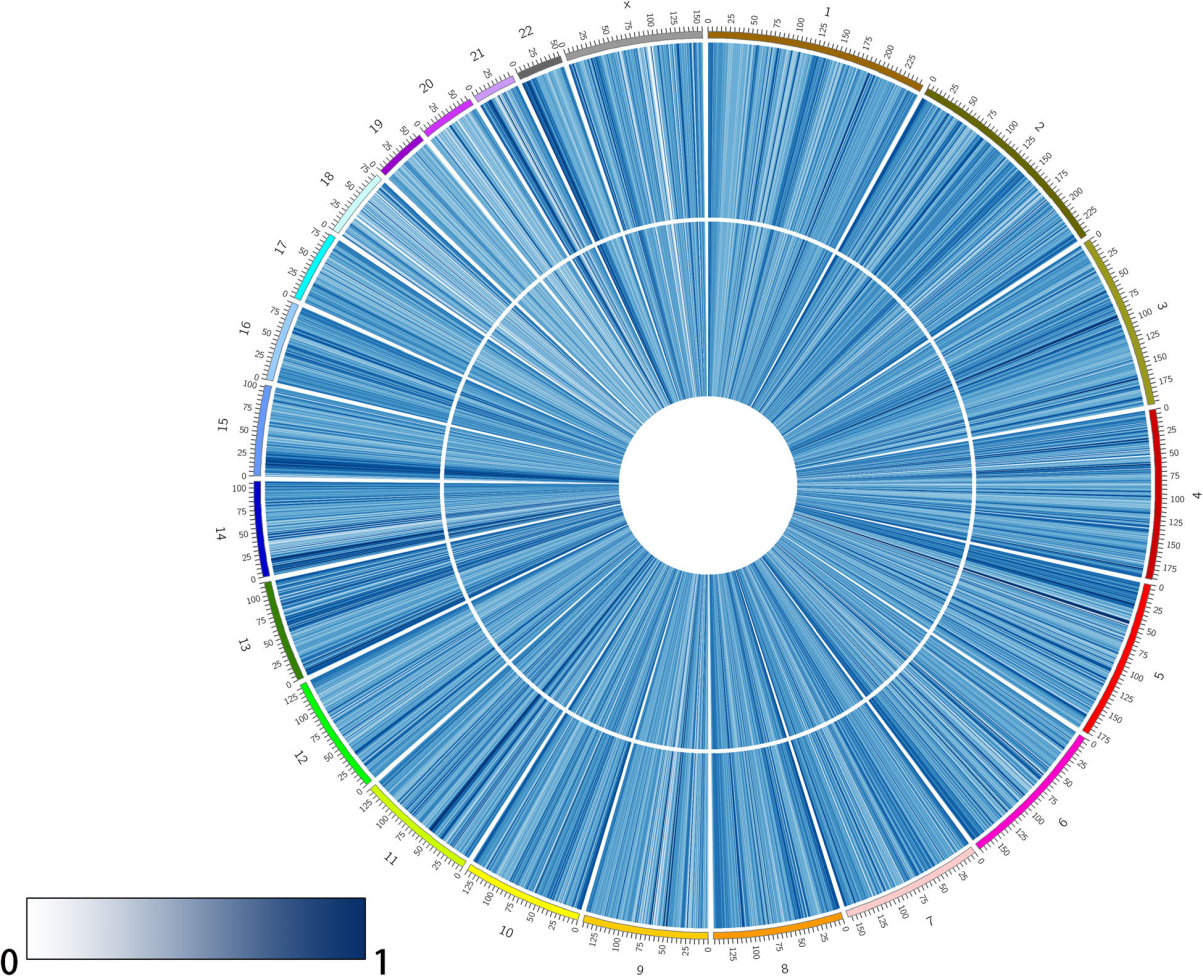
Overview of the average genome-wide DNA methylation level of genomic loci at each chromosome in Hainan centenarians and healthy controls, analyzed by Circos software. The inner and outer tracks represent the average methylation level for centenarians and healthy controls, respectively. All chromosomes are presented as 10Mbp-wide windows. The average methylation level in each region is represented as the average β-value (0–1) for all of the probes in that region

**Fig. 2:**
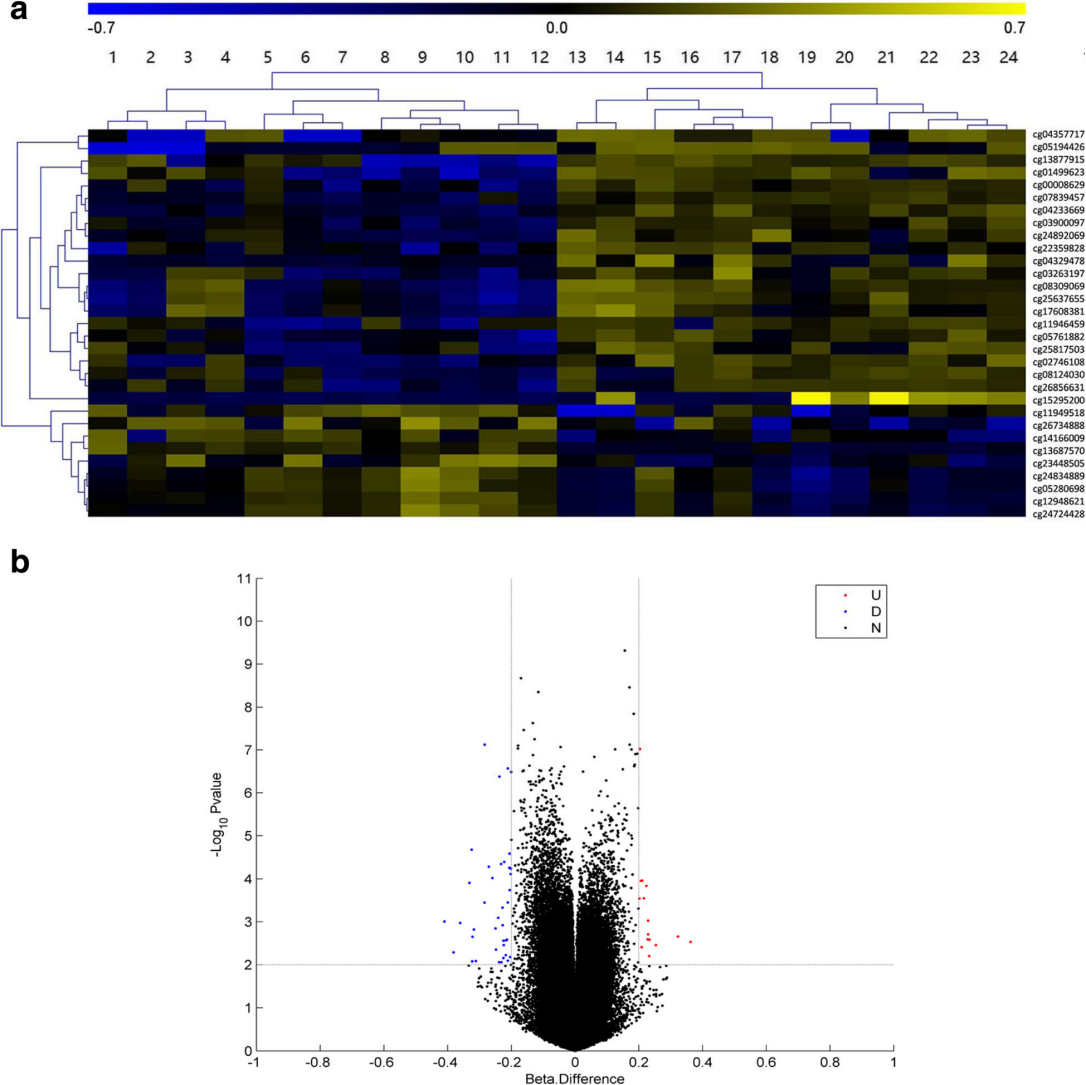
**a** Heatmap shows a clustering analysis of the 31 CpG sites that were significantly differentially methylated between Hainan centenarians and healthy controls. Columns: individual samples (1–12 are centenarians; 13–24 are healthy controls). Rows: CpG sites. Blue color: low methylation level; yellow color: high methylation level. **b** Volcano plot: each dot on the plot is a single CpG site. Horizontal axis: Beta. Difference (Δβ); vertical axis: -log10 of *p* value. Colour coding is based on the Δβ. Thick vertical lines highlight Δβ of − 0.2 and 0.2, while a thick horizontal line represents a *p*-value of 0.01. The red dots and blue dots represent hypermethylated sites and hypomethylated sites in centenarians, respectively

Using microarray and pyrosequencing methods, we performed an analysis of DNA methylation patterns in centenarians and healthy controls from Hainan Province. An overview of the genome-wide methylation profiles for both groups is shown in Fig. 1. Different patterns of methylation between centenarians and healthy controls were observed at the majority of methylation loci, indicating that DNA methylation-based epigenetic profiles might be significant in aging. Having discovered these differences in DNA methylation, we then searched for individual DMPs. To do this, we performed a pooled-test to analyze the average methylation level at all of the CpG loci in the centenarian and healthy control groups. In total, 31 DMPs were identified using the criteria *P* < 0.01 and |Δ*β*| > 0.2, as shown in Fig. 2a and b. In this analysis, Δ*β* > 0.2 and Δ*β* < − 0.2 refer to loci that were hypermethylated or hypomethylated in centenarians relative to healthy controls, respectively. Among the 31 DMPs identified, 9 were hypermethylated CpG sites and 22 were hypomethylated CpG sites (Table 2).

**Table 2:** List of 31 CpG sites showing significant differences in methylation between centenarians and healthy controls

Probe ID	*P* Value	FDR	Δ *β*	Gene	Region	Chromosome
Hypermethylated genes						
cg24724428	9.49E-08	2.94E-03	0.203271068	ELOVL2	TSS1500	6
cg23448505	1.09E-04	6.99E-02	0.210419612	HKR1	5’UTR	19
cg14166009	1.10E-04	7.04E-02	0.206571283	HKR1	TSS1500	19
cg13687570	1.46E-04	7.97E-02	0.224156561	HKR1	TSS1500	19
cg26734888	2.82E-04	1.08E-01	0.215848099	HKR1	TSS1500	19
cg12948621	2.86E-04	1.09E-01	0.201660605	HKR1	TSS200	19
cg24834889	9.41E-04	1.79E-01	0.229230299	HKR1	TSS200	19
cg05280698	1.95E-03	2.32E-01	0.22951704	HKR1	TSS200	19
cg11949518	2.54E-03	2.52E-01	0.227662305	RPTOR	Body	17
Hypomethylated genes						
cg07839457	7.52E-08	2.94E-03	−0.283566443	NLRC5	TSS1500	16
cg04233669	2.70E-07	4.49E-03	−0.210848478	LOC153328	Body	5
cg00008629	4.19E-07	5.21E-03	−0.237495141	ROD1	5’UTR	9
cg24892069	2.60E-05	2.07E-01	−0.205487552	NRP1	TSS1500	10
cg01499623	4.50E-05	4.55E-02	−0.232050941	SMU1	Body	9
cg22359828	5.49E-05	5.01E-02	−0.206320371	CACNA1E	Body	1
cg03930097	7.66E-05	5.82E-02	−0.201870989	HLA-DRA	Body	6
cg04329478	9.57E-05	6.52E-02	−0.259320244	LOC400696	TSS200	19
cg17608381	1.24E-04	7.37E-02	−0.331606794	HLA-A	Body	6
cg04357717	3.55E-04	1.21E-01	−0.210909327	ZPLD1	Body	3
cg11946459	3.56E-04	1.21E-01	−0.283888936	HLA-A	Body	6
cg13877915	8.02E-04	1.68E-01	−0.241392036	ZNF132	TSS200	19
cg26856631	9.82E-04	1.81E-01	−0.409805022	LSM5	3’UTR	7
cg25817503	1.20E-03	1.95E-01	−0.227151237	AFAP1	Body	4
cg08309069	1.43E-03	2.07E-01	−0.249630086	HLA-C	TSS1500	6
cg08124030	2.23E-03	2.41E-01	−0.321896757	TM4SF1	1stExon	3
cg05761882	2.59E-03	2.53E-01	−0.213920774	CAPN2	Body	1
cg15295200	5.13E-03	3.14E-01	−0.381483294	NMNAT3	TSS1500	3
cg25637655	5.97E-03	3.28E-01	−0.217322003	HLA-A	Body	6
cg03263197	6.62E-03	3.36E-01	−0.203535875	UBL4B	TSS1500	1
cg02746108	6.97E-03	3.40E-01	−0.225251292	AGAP1	Body	2
cg05194426	8.66E-03	3.59E-01	−0.230652944	CYP2E1	Body	10

### GO and KEGG functional enrichment analysis of differentially methylated genes

Our results showed that the 31 identified significantly differentially methylated sites described above could be mapped to 23 different genes. We performed GO term and KEGG pathway enrichment analyses to elucidate the biological functions of these genes, using the R package GOstats described previously [32]. The biological processes, molecular functions, and cellular components that were enriched in the GO analysis of longevity-associated DMP-containing genes (DMGs) are summarized in Table 3. In total, 3 biological processes, 6 cellular components, and 2 molecular function terms were enriched in the 23 DMGs. The enriched biological functions, such as antigen processing and presentation of peptide antigen (GO: 0048002) are associated with immune system function. Furthermore, enriched cellular components include major histocompatibility complex (MHC) protein complex and insoluble fraction (GO: 0042611 and GO: 0005626, respectively). MHC receptor activity, class I and II (GO: 0032393 and GO: 0032395, respectively), are enriched molecular functions in the DMG subset.

**Table 3:** Analysis of significantly hypermethylated differentially methylated probes (DMPs)

GO ID	Description	*P* Value	Genes
Biological process			
GO:0048002	antigen processing and presentation of peptide antigen	4.27E-04	HLA-A, HLA-C, HLA-DRA
GO:0019882	antigen processing and presentation	3.70E-03	HLA-A, HLA-C, HLA-DRA
GO:0002474	antigen processing and presentation of peptide antigen via MHC class I	1.86E-02	HLA-A, HLA-C
Cellular component			
GO:0042611	MHC protein complex	1.71E-03	HLA-A, HLA-C, HLA-DRA
GO:0042612	MHC class I protein complex	3.02E-02	HLA-A, HLA-C
GO:0005626	insoluble fraction	5.95E-02	ELOVL2, HLA-C, CYP2E1, CAPN2
GO:0044459	plasma membrane part	7.76E-02	HLA-A, HLA-C, CACNA1E, TM4SF1, AFAP1, HLA-DRA
GO:0005886	plasma membrane	8.86E-02	NRP1, HLA-A, HLA-C, CACNA1E, TM4SF1, CAPN2, AFAP1, HLA-DRA
GO:0031224	intrinsic to membrane	8.97E-02	ZPLD1, NRP1, ELOVL2, HLA-A, HLA-C, CACNA1E, TM4SF1, LOC153328, CYP2E1, HLA-DRA
Molecular function			
GO:0032393	MHC class I receptor activity	2.07E-02	HLA-A, HLA-C
GO:0032395	MHC class II receptor activity	2.31E-02	HLA-C, HLA-DRA

KEGG analysis revealed the enrichment of only several pathways in the DMG subset (Table 4), with the most significantly enriched biological pathways being graft-versus-host disease (KEGG: 05332) and allograft rejection (KEGG: 05330).

**Table 4:** KEGG pathway enrichment analysis of significantly differentially methylated probes (DMPs)

KEGG ID	Description	*P* Value	Genes
KEGG:05332	Graft-versus-host disease	1.18E-03	HLA-A, HLA-C, HLA-DRA
KEGG:05330	Allograft rejection	1.48E-03	HLA-A, HLA-C, HLA-DRA
KEGG:04940	Type I diabetes mellitus	1.91E-03	HLA-A, HLA-C, HLA-DRA
KEGG:05320	Autoimmune thyroid disease	2.92E-03	HLA-A, HLA-C, HLA-DRA
KEGG:05416	Viral myocarditis	3.50E-03	HLA-A, HLA-C, HLA-DRA
KEGG:04612	Antigen processing and presentation	6.15E-03	HLA-A, HLA-C, HLA-DRA
KEGG:05166	HTLV-I infection	6.64E-03	NRP1, HLA-A, HLA-C, HLA-DRA

### Changes in ROD1, NLRC5 and HKR1 DNA methylation in centenarians

The CpGs associated with aging were not uniformly distributed across chromosomes, CpG islands, or genes; significantly differentially methylated sites were over-represented on chromosomes 3, 6, 9, and 19 (Table 2).

**Fig. 3:**
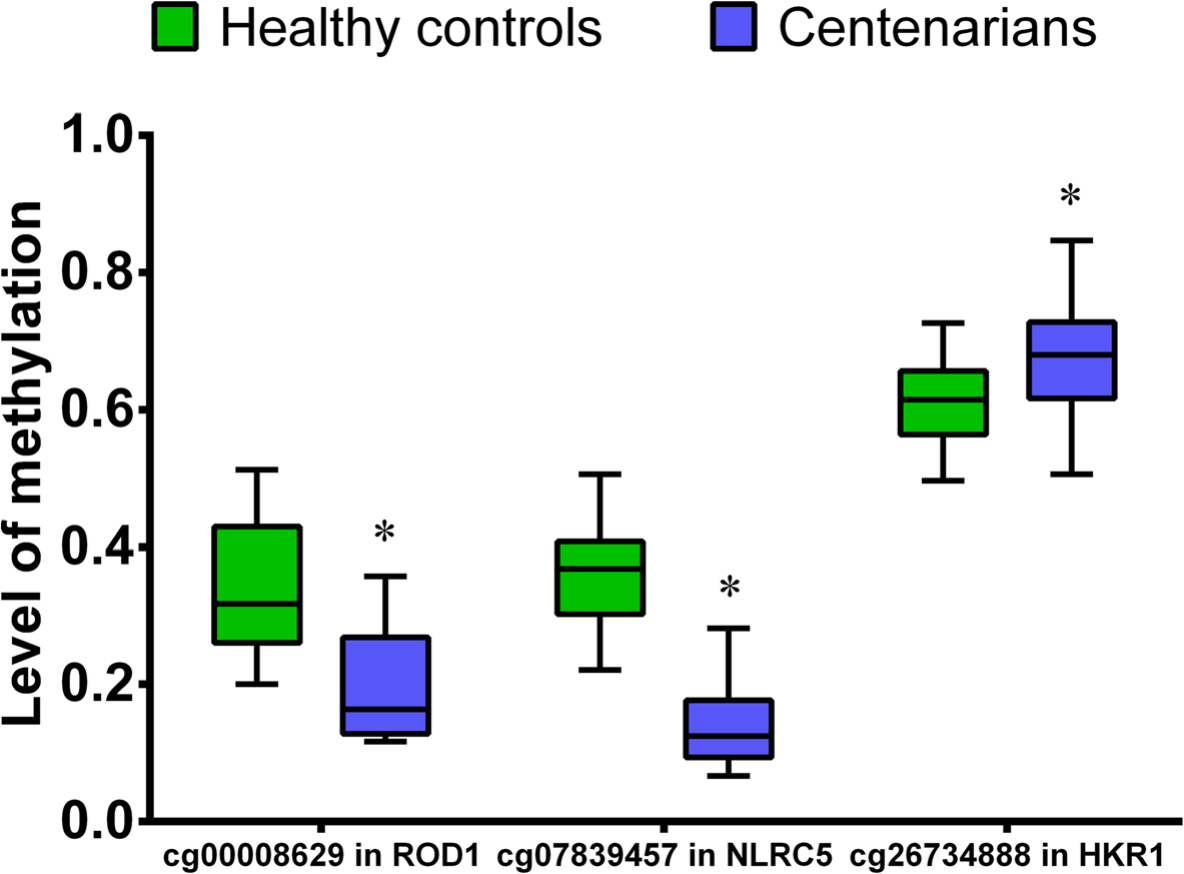
The level of methylation at the indicated CpG sites in centenarians (blue boxes) and healthy controls (green boxes). The boxes represent the 25th to the 75th centiles, and solid lines represent the median value for each sample. Error bars represent 95% confidence intervals. * *p* < 0.05 indicated the significant different between healthy controls and centenarians

The gene locations of the DMPs were assessed, and defined as follows: 1500 bp upstream of the TSS (TSS1500), 200 bp upstream of the TSS (TSS200), the 5’-UTR, exon 1, the gene body (encompassing all exons except exon 1), and the 3’-UTR. Some of the enriched DMPs were located within the gene body, but the role of methylation within the gene body is less clear. DNA methylation of promoter regions is a repressive epigenetic mark that regulates gene expression. The three most significantly hypermethylated genes in the centenarian group were ELOVL fatty acid elongase 2 (ELOVL2; ID cg24724428), histidine kinase rhodopsins 1 (HKR1; ID cg23448505; ID cg14166009; ID cg13687570; ID cg26734888; ID cg12948621; ID cg24834889; IDcg05280698) and regulatory associated protein of MTOR complex 1 (RPTOR; ID cg11949518). The CpG islands in the ELOVL2 gene was located within the gene promoter (TSS1500), HKR1 gene was located within TSS1500, TSS200 and 5’-UTR, while RPTOR genes were located within the gene body. Previous studies demonstrated that methylation of a CpG island within the ELOVL2 gene strongly correlates with age; indeed such methylation was declared a novel epigenetic marker for aging [33, 34]. Furthermore, seven gene sites (ID cg23448505; ID cg14166009; ID cg13687570; ID cg26734888; ID cg12948621; ID cg24834889; IDcg05280698) belong to the same gene(HKR1), therefore, we chose one of HKR (ID cg26734888) to pyrosequence in a large sample set. The three most significantly hypomethylated genes in the centenarians were NLR family CARD domain containing 5 (NLRC5; ID cg07839457), regulator of differentiation 1 (ROD1; ID cg00008629) and LOC153328 (ID cg04233669). The CpG islands in the NLRC5 gene was located TSS1500; ROD1 gene was located 5’UTR, while LOC153328 gene was located within the gene body. It has been reported that the methylation level of CpGs within the coding region can regulate gene transcription [35]. To confirm these observations, we chose NLRC5 (ID cg07839457) and ROD1 (ID cg00008629) genes to pyrosequence in a large sample set. In total, 60 peripheral blood samples (30 samples from each group) underwent pyrosequencing. As expected, significant differences in the level of methylation at the sites cg00008629 (ROD1), cg07839457 (NLRC5), and cg26734888 (HKR1) were observed between Hainan centenarians and healthy controls (Table 5 and Fig. 3). Using a regression-based approach, we further analyzed the DNA methylation data based on cell type compositions [36]. Our further analyses indicated that the differences in the level of methylation of cg00008629 (ROD1), cg07839457 (NLRC5), and cg26734888 (HKR1) sites were significant (*p* value < 0.05) between centenarians and healthy controls (Table 6).

**Table 5:** List of CpG sites showing significant differences between Hainan centenarians and healthy controls

Site	Gene	T	*P*
cg00008629	ROD1	−6.388	<0.001
cg07839457	NLRC5	−12.779	<0.001
cg26734888	HKR1	3.363	0.001

**Table 6:** List of 3 CpG sites showing significant differences in methylation between centenarians and healthy controls after adjusted for cell type composition in DNA methylation data

Site	Gene	*P* (before cell type correction)	*P* (after cell type correction)
cg00008629	ROD1	4.19E-07	3.48 E-02
cg07839457	NLRC5	7.52E-08	1.99 E-02
cg26734888	HKR1	2.82E-04	4.46 E-02

## Discussion

In our current study, we analyzed theDNA methylation of blood samples collected from centenarians and healthy controls in Hainan province, China. In summary, we found 31 DMPs using a 450 K array. Furthermore, our pyrosequencing analysis showed that methylation level at three of CpGs, ROD1, NLRC5, and HKR1 strongly correlates with aging in centenarians.

Our results also indicate that 31 high-confidence CpG sites with differential methylation profiles between the groups; 9 (29%) were hypermethylated and 22 (71%) were hypomethylated in centenarians. KEGG pathway analysis showed that the most significantly enriched biological pathways being graft-versus-host disease and allograft rejection. Further studies are needed to study how these pathways relate to the aging exhibited by centenarians.

The ROD1 gene was discovered to be hypomethylated in centenarians. This gene encodes an RNA-binding protein that binds preferentially to poly(G) and poly(U) sequences in vitro, and was initially regarded as an inhibitor of differentiation [37]. Several ROD1 transcriptional variants, encoding different isoforms, have been identified. In addition, ROD1 was recently found to be a member of the heterogeneous nuclear ribonucleoprotein family, which regulate the alternative splicing of pre-RNA, and can thus modulate gene expression by repressing the expression of exons in a tissue specific manner [38, 39]. Further, ROD1 binding to C-terminal peptide was strongly inhibited by synthetic RNA and weakly inhibited by a synthetic phosphorylated peptide designed to mimic the C-terminal domain of RNA polymerase II. Interestingly, ROD1 expression and knockdown experiments suggest a role in the regulation of C-terminal peptide mitogenic activity [40], suggesting a potential correlation between hypomethylation of ROD1 gene and aging in centenarians.

NLRC5, a member of the NOD-like receptor gene family and widely expressed in tumor tissues, is a key transcriptional regulator of MHC class I molecules [41, 42]. Cancers can escape immune surveillance by reducing the expression of MHC class I molecules. NLRC5, an important regulator of innate immune responses, may affect patient survival by regulating this immune evasion via MHC class I molecules. NLRC5 could therefore be exploited to restore tumor immunogenicity and to stimulate protective antitumor immunity [43]. While the role of NLRC5 in both innate and adaptive immune signaling is well established, its function in inflammation remains poorly understood. The regulation of NF-κB activation is crucial for many biological functions, and NF-κB dysregulation is associated with immune deficiency, infectious disease, inflammation, and cancer [44, 45]. NLRC5 has been reported to inhibit NF-κB activation by interacting with IKK-α/−β and blocking its phosphorylation [46]. To date, there have been no reports which implicate NLRC5 in aging, however, we found that NLRC5 was hypomethylated in centenarians. Methylation of the NLRC5 is negatively correlated with NLRC5 expression in many cells [47, 48]. It is possible that this NLRC5 hypomethylation is involved in aging through the regulation of various biological pathways.

In contrast to ROD1 and NLRC5, HKR1 was hypermethylated in centenarians. HKR1 encodes Krueppel-related zinc finger protein 1, a member of the GLI-Kruppel zinc finger family that is important for gene regulation [49]. The HKR1 protein possesses a serine/threonine-rich domain, and a Ca^2+^-binding consensus sequence within its cytoplasmic domain. Recently, HKR1 mRNA expressions were found to be higher in lung cancer tissues than in normal lung tissues, and HKR1 may be involved in the regulation of a signaling pathway involved in lung cancer progression [50]. Although the physiological roles of HKR1 remain unclear, HKR1 knockout is lethal, indicating that this gene is essential for survival. Further research is needed to fully understand the significance of HKR1 hypermethylation in centenarians and its effect on aging.

However, it is worth mentioning that peripheral blood is a heterogeneous collection of different cell types, each with a very different DNA methylation profile. Differences in the relative proportions of these components may contribute to differences in DNA methylation [51–53]. To identify the potential effect of cellular proportions in the DNA methylation differentiation, we used a regression-based approach to adjusting for cell type composition in DNA methylation data. Our results indicated that the methylation cg00008629 (ROD1), cg07839457 (NLRC5), and cg26734888 (HKR1) were also found significant differences between centenarians and healthy controls.

To our knowledge, the association between the genes ROD1, NLRC5, and HKR1 and aging have not been previously reported. We here found significantly different levels of DNA methylation at the ROD1, NLRC5, and HKR1 genes in centenarians and healthy controls. Our findings implicate that these genes may play important roles in aging via the regulation of various biological pathways and could be used as biomarker genes for aging.

## Conclusions

In summary, we present a comprehensive comparison of genomic methylation in centenarians and healthy controls residing in Hainan Province. We report an epigenetic signature consisting of three genes, which can be used to estimate aging from blood samples. Changes to the methylation of CpG islands, namely, hypomethylation within the ROD1 and NLRC5 and hypermethylation within HKR1 genes, are possibly correlated with aging in centenarians. While further studies are required to understand the mechanisms and signaling pathways through which ROD1, NLRC5, and HKR1 methylation affect aging, the methylation of these genes constitutes a signature that could be used to evaluate aging. The combination of ROD1, NLRC5, and HKR1 gene methylation is therefore a potential novel biomarker for aging.

## Additional file

Additional file 1: “Pyrosequencing primer sequences of three sites”. This file contains detailed description of pyrosequencing primer sequences. (DOCX 12 kb)

## Abbreviations

CpG: cytosine-phosphate-guanosine
DMG: DMP-containing genes
DMP: Differentially methylated probe
ELOVL2: ELOVL fatty acid elongase 2
GO: Gene ontology
HKR1: Histidine kinase rhodopsins 1
KEGG: Kyoto Encyclopedia of Genes and Genomes
MHC: Major histocompatibility complex
NLRC5: NLR family CARD domain containing 5
ROD1: Regulator of differentiation 1
TSS: Transcription start sites
UTR: Untranslated regions
