# Supplementary material for: Methylation of the genes ROD1, NLRC5, and HKR1 is associated with aging in Hainan centenarians
Source: BMC Med Genomics. 2018 Feb 2;11:7. doi: 10.1186/s12920-018-0334-1 (PMC5797414; doi:10.1186/s12920-018-0334-1)
Supplement: Additional file 1: — “Pyrosequencing primer sequences of three sites”. This file contains detailed description of pyrosequencing primer sequences. (DOCX 12 kb) [file 12920_2018_334_MOESM1_ESM.docx]

**Pyrosequencing primer sequences of three sites:**

cg00008629 (ROD1),

forward PCR Primer: TGATTTTTATGAGGTAATGATGATATAGG

reverse PCR Primer: TATAAAAATCCCCTTTAAAATCCTACCAT

sequencing Primer: TTTTTAGGAGATATGAAGAAAAATA

cg07839457 (NLRC5),

forward PCR Primer: TAGGGGAGAAGGGAATGGT

reverse PCR Primer: CTTTCCATCTCCCCCTTTC

sequencing Primer: AGAAGGGAATGGTAGTA

cg26734888 (HKR1),

forward PCR Primer: GTAGAGTATAGGGATTATATTTTGTAGAA

reverse PCR Primer: ACCCACTCTTAAAATACCCAAATA

sequencing Primer: GTATAGGGATTATATTTTGTAGAAT
